# The multifaceted role of macrophages in kidney physiology and diseases

**DOI:** 10.3389/fimmu.2025.1642525

**Published:** 2025-09-19

**Authors:** Yiqi Ma, Fengning Yang, Jingjuan Yang, Kewu Wang, Jibo Hu, Qian Wu

**Affiliations:** ^1^ Department of Radiology, Center for Regeneration and Aging Medicine, the Fourth Affiliated Hospital of School of Medicine, and International School of Medicine, International Institutes of Medicine, Zhejiang University, Yiwu, Zhejiang, China; ^2^ School of Medical Imaging, Hangzhou Medical College, Hangzhou, China; ^3^ Department of Nephrology, Center for Regeneration and Aging Medicine, the Fourth Affiliated Hospital of School of Medicine, and International School of Medicine, International Institutes of Medicine, Zhejiang University, Zhejiang-Denmark Joint Laboratory of Regeneration and Aging Medicine, Yiwu, Zhejiang, China

**Keywords:** macrophage heterogeneity, kidney physiology, acute kidney injury, chronic kidney disease, sing-cell

## Abstract

Macrophages are highly plastic and heterogeneous innate immune cells that play pivotal roles in kidney development, kidney functions maintenance, immune surveillance, injury, repair, fibrosis and so on. Our understanding of embryonic derived and bone marrow–derived macrophages has evolved beyond the classical M1/M2 polarization paradigm, shifting toward a more nuanced investigation of macrophage subpopulations through the lens of functional specialization and tissue-specific adaptation. Recent advancements in single-cell and spatial transcriptomics have elucidated the diversity of kidney macrophages, revealing their critical contribution to kidney physiology and pathology. In acute kidney injury, macrophages orchestrate inflammatory cascades via cytokine secretion and inflammasome activation, whereas during the reparative phase, they promote tissue regeneration through anti-inflammatory pathways. However, persistent or dysregulated macrophage activation can lead to maladaptive repair and progression to chronic kidney disease characterized by kidney fibrosis. Therapeutically, targeting macrophage polarization, recruitment and macrophage-based adoptive cell therapy has emerged as a promising strategy for modulating kidney inflammation and fibrosis. This review delineates the multifaceted roles of diverse macrophage subsets in kidney physiology and pathology, while highlighting emerging therapeutic avenues and the translational challenges associated with macrophage-targeted interventions.

## 1 Introduction

Macrophages are a heterogeneous population integral to the phagocytic system. While initially identified for their role in eliminating infectious agents, they are now recognized as key regulators of pro- and anti-inflammatory responses, tumor microenvironment dynamics, tissue repair and fibrosis, and systemic metabolic processes—functions that have garnered growing attention for their critical involvement in kidney physiology and pathology.

Kidney macrophages represent an indispensable cellular population within the kidney, distinguished by marked phenotypic heterogeneity and remarkable plasticity (1). Owing to this adaptability, they assume distinct functional phenotypes across states of homeostasis, injury, and repair (2, 3). However, the functional attributes and mechanistic foundations of specific kidney macrophage subsets remain incompletely defined, underscoring a critical need for continued investigation.

Advancements in technologies such as single-cell and spatial transcriptomics have markedly accelerated our understanding of kidney macrophages in recent years (4, 5), enabling comprehensive delineation of their intricate regulatory networks. This review seeks to elucidate the multifaceted roles of heterogeneous macrophage subsets in kidney physiology and pathology and to explore their prospective implications for therapeutic intervention in kidney diseases.

## 2 Macrophage biology and heterogeneity in the kidney

Macrophage heterogeneity is an intrinsically complex subject, with diverse interpretations yielding distinct contextual frameworks. This section delineates the concept through three principal dimensions: ontogeny, polarization, and function, and their implications in kidney macrophage. We then provide the molecular signature of kidney macrophage at the end of this section.

### 2.1 Ontogeny

Although macrophages were initially thought to arise predominantly from hematopoietic sources, this notion has been challenged over recent decades (6). Current evidence indicates that macrophage populations in various tissues and developmental stages derive from three distinct ontogenetic waves: primitive yolk sac–derived macrophages, fetal liver–derived monocytes, and bone marrow–derived monocytes.

Embryonic macrophages encompass both early yolk sac–derived macrophages and fetal liver–derived monocytes (7). In mouse models, the first wave originates from yolk sac erythro-myeloid progenitors (EMPs) at embryonic day 7.0 (E7.0). A subsequent wave of EMPs emerges around E8.5, migrates to the fetal liver, and differentiates into EMP-derived macrophages (8). These cells, often termed “pre-macrophages,” undergo rapid phenotypic diversification upon colonizing target tissues. Although EMP-derived macrophages initially populate embryonic tissues, they are progressively replaced by fetal liver–derived monocytes. By E10.5, hematopoietic stem cells (HSCs) from the aorta–gonad–mesonephros (AGM) region seed the fetal liver and later colonize the bone marrow by E17.5. Postnatally, monocyte-derived macrophages (MDMs) are maintained in each organ through continual replenishment by circulating HSC-derived monocytes (9, 10).

Kidney macrophages consist of both embryo-derived and monocyte-derived populations, with embryo-derived macrophages constituting the predominant subset. The proportions of embryo-derived macrophages within the total kidney macrophage population were 7.27 ± 0.4%, 46.7 ± 2.5%, and 98.8 ± 0.3% following induction at E8.5, E13.5, and E18.5, respectively (11). After birth, embryo-derived macrophages are maintained primarily through self-renewal mediated by the CX3C chemokine receptor 1 (CX3CR1)/CX3CL1 axis (12). Embryo-derived macrophages, which constitute the majority of kidney macrophages and serve as kidney resident macrophages (KRMs), together with MDMs, form the kidney macrophage compartment and cooperatively sustain kidney homeostasis. Under physiological conditions, the proportion of kidney macrophages derived from monocyte-derived macrophages (MDMs) progressively increases with age, reaching approximately 40% (11). In an adoptive transfer mouse model and irradiation, MDMs have the capacity to differentiate into KRMs, thereby preserving the integrity of the kidney niche (11, 13).

### 2.2 Polarization

Earlier investigations have extensively examined macrophage heterogeneity and function through the conceptual framework of polarization—a paradigm that has become classical and remains widely adopted.

Macrophage differentiation has been broadly categorized into classical M1-type and alternative M2-type activation, mirroring the T helper 1 (TH1) and T helper 2 (TH2) paradigms of T cell differentiation (3, 14).

M1 polarization is driven by pathogen-associated molecular patterns (PAMPs), damage-associated molecular patterns (DAMPs), and pro-inflammatory cytokines—particularly Th1-derived cytokines such as tumor necrosis factor-α (TNF-α) and interferon-γ (IFN-γ) (15). Hallmarks of M1 polarization include the upregulation of surface activation markers and antigen presentation molecules, including major histocompatibility complex (MHC) class II, CD16, CD32, CD80, CD86, secreted phosphoprotein 1 (SPP1), and the interleukin (IL)-1 receptor (IL-1R). M1 macrophages also produce a diverse repertoire of pro-inflammatory mediators, such as IL-1, IL-6, IL-12, IL-23, inducible nitric oxide synthase (iNOS), matrix metalloproteinase-12 (MMP-12), and macrophage-inducible C-type lectin (MINCLE) (3, 16), all of which contribute to inflammatory processes.

In contrast, M2 macrophages exert diverse functions in immune regulation, notably in suppressing inflammation and promoting tissue repair. M2 polarization is driven by Th2-derived cytokines such as IL-4, IL-10, and transforming growth factor-β (TGF-β) (15). M2 macrophages are commonly identified by the expression of markers such as arginase 1 (Arg1), the mannose receptor (CD206), ferroportin 1 (FPN1, SLC40A1), and TGF-β (2, 17). They are further subclassified into four phenotypically and functionally distinct subtypes: M2a, M2b, M2c, and M2d (3, 18).

The classical M1/M2 polarization model remains widely employed in describing kidney macrophages. In the early stages of kidney injury, M1 macrophages predominate, functioning to clear apoptotic cells. Furthermore, M1 macrophages, together with T cells and dendritic cells (DCs), contribute to the formation of lymphoid aggregates, thereby establishing a pro-inflammatory microenvironment that amplifies kidney inflammatory responses (19). As the disease progresses into the reparative phase, M2 macrophages become the dominant subset, exerting anti-inflammatory and pro-repair effects (20). However, when tissue repair fails, M2 macrophages can also acquire pro-fibrotic properties.

Current perspectives posit that macrophages exist along a continuum of phenotypes encompassing M1-like, M2-like, and mixed states (21–23).

### 2.3 Function

Macrophages should not be viewed merely as binary responders to polarizing cues. Instead, in response to specific microenvironmental stimuli, they possess the capacity to differentiate into diverse subpopulations equipped to perform highly specialized functions (24, 25).

Perivascular macrophages (PVMs) reside adjacent to blood vessels (26), where they regulate vascular permeability, maintain tissue homeostasis, and modulate endocrine function (26); they also act as a barrier against exogenous toxins but can promote pathological angiogenesis in tumors, facilitating metastasis (27–29). Iron-recycling macrophages, characterized by ferroportin 1 (FPN1) expression and rely on colony-stimulating factor 1 (CSF1) and the transcription factor (NRF2) (30), clear senescent or damaged erythrocytes to maintain systemic and cellular iron homeostasis (31). Sympathetic neuron–associated macrophages (SAMs), marked by solute carrier family 6 member 2 (SLC6A2) and Monoamine Oxidase A (MAOA) expression, metabolize norepinephrine in the sympathetic nervous system and influence thermogenesis, with presence confirmed in human sympathetic ganglia (32). Lipid-associated macrophages (LAMs), enriched in metabolic disorders such as atherosclerosis, nonalcoholic steatohepatitis (NASH), and obesity (33), are specialized in lipid handling and apoptotic cell clearance (34–37), but may also potentiate inflammation and aggravate disease progression (38, 39). Efferocytic macrophages specialize in apoptotic cell clearance via receptor-mediated or bridging molecule–mediated recognition, leveraging apoptotic cell–derived nucleotides to boost phagocytic activity, thereby reducing apoptotic burden and preventing secondary tissue injury (40, 41).

Several of the aforementioned functional types of macrophages are identified in the kidney. Researchers found a subset of embryonically derived KRMs (phenotypically similar to SAM) that localize in close proximity to kidney sympathetic nerves and are indispensable for maintaining sympathetic innervation, and highly express sympathetic nerve–relevant genes including MAOA, Neuropeptide Y receptor type 1 (Npy1r), and SLC6A2 (42). Patients with ANCA-associated glomerulonephritis (AGN) have a marked enrichment of SPP1^+^ LAMs within the kidney, where they contribute to both pro-inflammatory and pro-fibrotic processes (43). In addition, efferocytic capacity of macrophages is adversely associated with the progression of diabetic kidney disease (DKD), suggesting a protective role of efferocytic macrophages in DKD (23). Furthermore, manipulation of the labile iron pool in kidney macrophages mitigate CKD mice model, implying the involvement of iron recycling macrophage in CKD (44).

Together, examining macrophage heterogeneity through a functional lens provides a more refined understanding of their subpopulation-specific roles, advancing our mechanistic insight into their contributions in the kidney under both physiological and pathological conditions.

### 2.4 Molecular signature of kidney macrophages

Initially, MDMs (Cd11b^high^, F4/80^low^) and KRMs (Cd11b^low^, F4/80^high^) were distinguished on the basis of differential expression of CD11b and F4/80 (45). Subsequent studies identified CD64 and C-C chemokine receptor type 2 (CCR2) as complementary markers for KRMs and MDMs, respectively (29, 46). Investigators further refine the subclassification of kidney macrophage subsets. For example, Some investigators have classified kidney macrophages into three distinct subsets based on the expression of CX3CR1 and CD81 (CX3CR1^-^CD81^-^, CX3CR1^+^CD81^-^, and CX3CR1^+^CD81^+^) (47).

## 3 Macrophage functions in kidney

**Figure 1:**
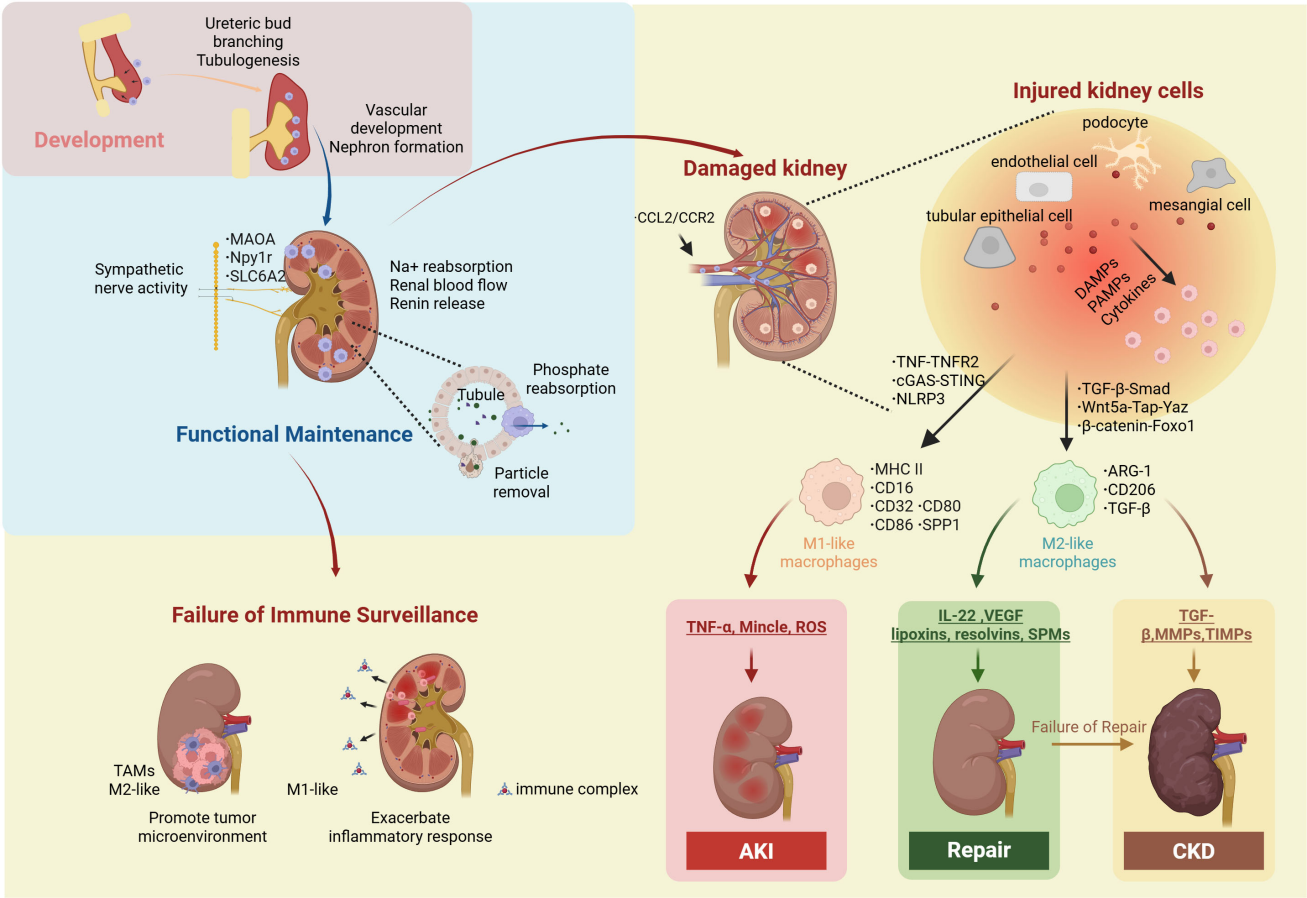
Macrophage in kidney. Kidney macrophages play essential roles in kidney development, kidney functional maintenance, immune surveillance, injury, repair, fibrosis. During development, they promote ureteric bud branching and tubulogenesis, thereby facilitating nephron formation and vascular development. In the adult kidney, macrophages express highly MAOA, Npy1r and SLC6A2 to maintain Na^+^ reabsorption, renin release, and renal blood flow by supporting the structural and functional integrity of sympathetic nerve fibers. In addition, they contribute to phosphate reabsorption and the preservation of tubular integrity and function through particle removal. When immune surveillance fails, tumor-associated macrophages (TAMs) contribute to the remodeling of the tumor microenvironment that promotes disease progression; meanwhile, kidney macrophages, influenced by factors such as immune complexes and *Escherichia coli*, can elicit excessive inflammatory responses. Injured renal cells (tubular epithelial cells, endothelial cells, podocytes, and mesangial cells) recruit circulating macrophages via the CCL2/CCR2 axis. These injured cells released DAMPs, PAMPs, and cytokines activate both infiltrating and resident macrophages through TNF-TNFR2, cGAS-STING, and NLRP3 inducing a proinflammatory phenotype (MHC II^+^, CD16^+^, CD32^+^, CD80^+^, CD86^+^, SPP1^+^) that secretes TNF-α, Mincle, and ROS to promote AKI. Alternatively, macrophages may polarize via TGF-β–Smad, Wnt5a–Tap–YAP, and β-catenin–Foxo1 pathways into a reparative/fibrotic phenotype (ARG1^+^, CD206^+^, TGF-β^+^), releasing TGF-β, MMPs, and TIMPs to drive CKD, or IL-22, VEGF, EVs, lipoxins, resolvins, and SPMs to support tissue repair. Npy1r, Neuropeptide Y receptor type 1; MAOA, Monoamine Oxidase A; SLC6A2, solute carrier family 6 member 2; TAMs, tumor-associated macrophages; CCL2, The C-C motif chemokine ligand 2; CCR2, C-C chemokine receptor type 2; TNF, Th1-derived cytokines such as tumor necrosis factor; TNFR2, tumor necrosis factor receptor 2; cGAS, cyclic GMP-AMP synthase; STING, stimulator of interferon genes; NLRP3, NOD-like receptor family pyrin domain containing 3; MHC class II, major histocompatibility complex; SPP1, secreted phosphoprotein 1; TGF-β, transforming growth factor-β; Smad, Suppressor of Mothers Against Decapentaplegic; Wnt5a, Wnt family member 5A; Yap, Yes-associated protein; Taz, Transcriptional co-activator with PDZ-binding motif; Foxo1, β-catenin/Forkhead box protein O1; Arg1, arginase 1; TNF-α, tumor necrosis factor-α; Mincle, macrophage-inducible C-type lectin; ROS, reactive oxygen species; VEGF, Vascular Endothelial Growth Factor; SPMs, specialized pro-resolving lipid mediators; MMP, matrix metalloproteinase; TIMPs, tissue inhibitors of metalloproteinases.

After introducing the classification and definition of kidney macrophages, we further delineate their roles in kidney physiology and pathology, including kidney development, kidney functional maintenance, immune surveillance, injury, repair, fibrosis, and non-canonical functions (**Figure 1**).

### 3.1 Kidney development

Mouse studies suggest that the development of the kidney is dependent on the regulatory functions of macrophages. At E9.5-E10.5, yolk sac–derived macrophages actively promote ureteric bud branching and tubulogenesis through the CSF-1/CSF1R signaling pathway (48), and contribute to early nephron patterning by clearing excess rostral nephron progenitors, thereby facilitating precise tissue organization (49). At E11.5-E12.5, macrophage–endothelial interactions are vital for vascular development, supporting vasculogenesis, vascular anastomosis, and the stabilization of kidney vascular architecture (49). At E12, fetal liver–derived macrophages start to infiltrate the mesenchyme of the developing metanephros, contributing to the formation of nephron components within both the cortical and medullary regions, including the loop of Henle (49, 50). The postnatal expansion and maturation of kidney macrophages parallel kidney growth, suggesting that they may play a role in this process (11).

### 3.2 Kidney functional maintenance

Kidney macrophages are essential for maintaining the reabsorptive functions of the renal tubules. Sympathetic stimulation triggers the release of norepinephrine, which promotes sodium and water reabsorption while concurrently reducing kidney perfusion. Emerging evidence suggests that embryonic-derived macrophages are essential for maintaining the basal distribution and functionality of sympathetic fibers, thereby safeguarding against electrolyte imbalances and preventing disorders associated with excessive urinary excretion of Na^+^, K^+^, and Cl^-^ (42, 51). In addition, cortical macrophages in the kidney exhibit elevated expression of the phosphate transporter solute carrier family 34 member 1 (SLC34A1), enabling the active reabsorption of phosphate from the urinary filtrate and thereby mitigating the risk of urinary stone formation (52). The kidney tubular epithelium forms a tight barrier that segregates urinary solutes from the interstitium. Subepithelial macrophages, strategically positioned beneath the epithelial layer, can extend cellular protrusions across the epithelium to sample and monitor the contents of the urine. This dynamic surveillance mechanism facilitates the removal of particulate matter and contributes to tubular integrity and function (53).

### 3.3 Immune surveillance

Under physiological conditions, kidney macrophages act as immune sentinels, continuously conducting surveillance to maintain tissue homeostasis. Upon detection of PAMPs or DAMPs, macrophages rapidly recognize these cues via pattern recognition receptors, initiating downstream signaling cascades that shape the broader kidney immune landscape (2, 3). Perturbation of this surveillance system may precipitate a variety of pathological conditions. KRMs are capable of recognizing small immune complexes (SICs), thereby triggering excessive immune activation that culminates in a type III hypersensitivity reaction (29). In renal cell carcinoma (RCC), tumor-associated macrophages (TAMs) (predominantly M2-like) facilitate immune evasion by promoting immunosuppression and upregulating PD-L1 expression within the tumor microenvironment. In clear cell renal cell carcinoma (ccRCC), TAMs secrete IL-23, thereby exacerbating immune evasion (54). Kidney macrophages participate in host defense against exogenous pathogens, such as uropathogenic *Escherichia coli* (UPEC), by phagocytosis, yet their simultaneous suppression of adaptive immunity and potential to induce hyperinflammatory injury contribute to disease persistence and kidney tissue damage (55, 56).

### 3.4 Injury

During kidney injury, macrophages are either locally activated or arise from circulating monocytes recruited to the damaged tissue (57–60). The C-C motif chemokine ligand 2 (CCL2)/CCR2 signaling axis is traditionally recognized as a principal mechanism for monocyte recruitment, particularly in DKD, IgA nephropathy (IgAN), and ischemia–reperfusion injury (IRI) (61–64). The injured kidney establishes a pro-inflammatory microenvironment that activates macrophages (65). In glomerulonephritis, damaged endothelial cells promote the conversion of CCR2^+^ monocytes into proinflammatory macrophages through the TNF/tumor necrosis factor receptor 2 (TNFR2) signaling axis, thereby exacerbating the inflammatory response (62). Injured tubular epithelial cells release DAMPs, such as self-DNA, HMGB1, *etc.* to activate downstream innate immune response including the cyclic GMP-AMP synthase (cGAS)/stimulator of interferon genes (STING) pathway (66), the NOD-like receptor family pyrin domain containing 3 (NLRP3) inflammasome in macrophages (67, 68). Together, these processes promote the conversion of macrophages into a proinflammatory phenotype. Additionally, phosphoinositide 3-kinase (PI3K) may facilitate macrophage infiltration into the kidney, thereby exacerbating kidney injury and fibrosis (58, 69, 70).

Activated macrophages contribute to kidney injury via multiple mechanisms (57). They secrete pro-inflammatory cytokines, such as IL-1β and TNF-α, that induce apoptosis and necrosis in glomerular and tubular epithelial cells, impairing filtration and reabsorption (67, 71). They also produce cytotoxic molecules such as the macrophage-inducible C-type lectin (Mincle), which recognizes necrotic tubular cells and amplifies inflammatory cytokine production while impeding clearance of dead cells in IRI (72). This establishes a self-perpetuating inflammatory cycle, in which junctional adhesion molecule-like protein (JAML) plays a critical role (73). Moreover, macrophages generate reactive oxygen species (ROS), directly damaging kidney macromolecules and exacerbating tissue injury (74).

### 3.5 Repair

As kidney repair progresses, macrophages undergo phenotypic transition toward a pro-repair phenotype (20, 75) (76). Although macrophages are involved, tubular epithelial cells remain the principal compartment contribute to the clearance of cellular debris and apoptotic bodies within the damaged microenvironment (77, 78). In addition to their scavenging functions, macrophages secrete a range of cytokines and growth factors—including IL-22 and Vascular Endothelial Growth Factor (VEGF)—that support angiogenesis and epithelial regeneration (79, 80).

Furthermore, kidney macrophages express anti-inflammatory mediators such as lipoxins, resolvins, and specialized pro-resolving lipid mediators (SPMs), which actively promote inflammation resolution and tissue homeostasis (81). For example, Maresin—a macrophage-derived SPM—facilitates lipid mediator class switching from pro-inflammatory to pro-resolving profiles, thereby supporting kidney repair (82).

### 3.6 Fibrosis

Kidney macrophages can also participate in kidney fibrosis under pathological conditions (83–86). Under pro-fibrotic conditions, macrophages can adopt a fibrogenic phenotype, characterized by the secretion of TGF-β and VEGF, induction of epithelial-mesenchymal transition (EMT), and impaired mitophagy, which collectively promote the progression of fibrosis.

TGF-β not only promotes M2-like polarization via the Suppressor of Mothers Against Decapentaplegic (Smad) signaling axis but also induces macrophage-to-myofibroblast transition (MMT) (87). Excessive pro-fibrotic macrophage accumulation, fueled by persistent TGF-β/Smad activation, facilitates collagen and extracellular matrix (ECM) deposition, exacerbating fibrotic remodeling (88). In the UUO model, both Wnt family member 5A (Wnt5a)/Yes-associated protein (Yap)/Transcriptional co-activator with PDZ-binding motif (Taz) and β-catenin/Forkhead box protein O1 (Foxo1) signaling pathways have been shown to potentiate TGF-β-mediated fibrogenesis through macrophage-dependent mechanisms (89, 90).

Moreover, macrophages secrete exosomes that contain CD63 and tumor susceptibility gene 101 (TSG101) to promote the progression of fibrosis in DKD (91). Concomitantly, kidney macrophages promote kidney lymphangiogenesis by activating the VEGF-C/VEGFR3 axis, thereby contributing to fibrosis progression (92).

Macrophages also facilitate EMT in tubular epithelial cells, a key event in fibrogenesis whereby epithelial cells acquire mesenchymal traits (93). Additionally, macrophages support fibroblast survival by delivering platelet-derived growth factor (PDGF) ligands through direct cell–cell interactions (94–96). In the UUO model, macrophage recruitment is further sustained through MMP-9 derived from injured tubular endothelial cells, which also promotes EMT and accelerates fibrotic progression (97).

Disruption of phosphatase and tensin homolog-induced kinase1 (PINK1)/mitofusin-2 (MFN2)/Parkin signaling pathway impairs mitochondrial quality control, driving macrophage polarization toward a pro-fibrotic phenotype, thereby exacerbating kidney fibrosis (98). Moreover, similar findings have been observed in CKD patients with interstitial fibrosis and tubular atrophy (99), Dysregulation of the kidney macrophage-specific mitochondrial quality control mechanism exacerbates fibrosis, unequivocally underscoring its substantial potential in preventing the progression of kidney fibrosis.

Moreover, MMT has garnered considerable attention (100), as it plays a pivotal role in driving the progression of kidney fibrosis. Increasing evidence indicates that targeting MMT could be a therapeutic strategy to attenuate fibrosis (101, 102).

### 3.7 Non-canonical functions

**Figure 2:**
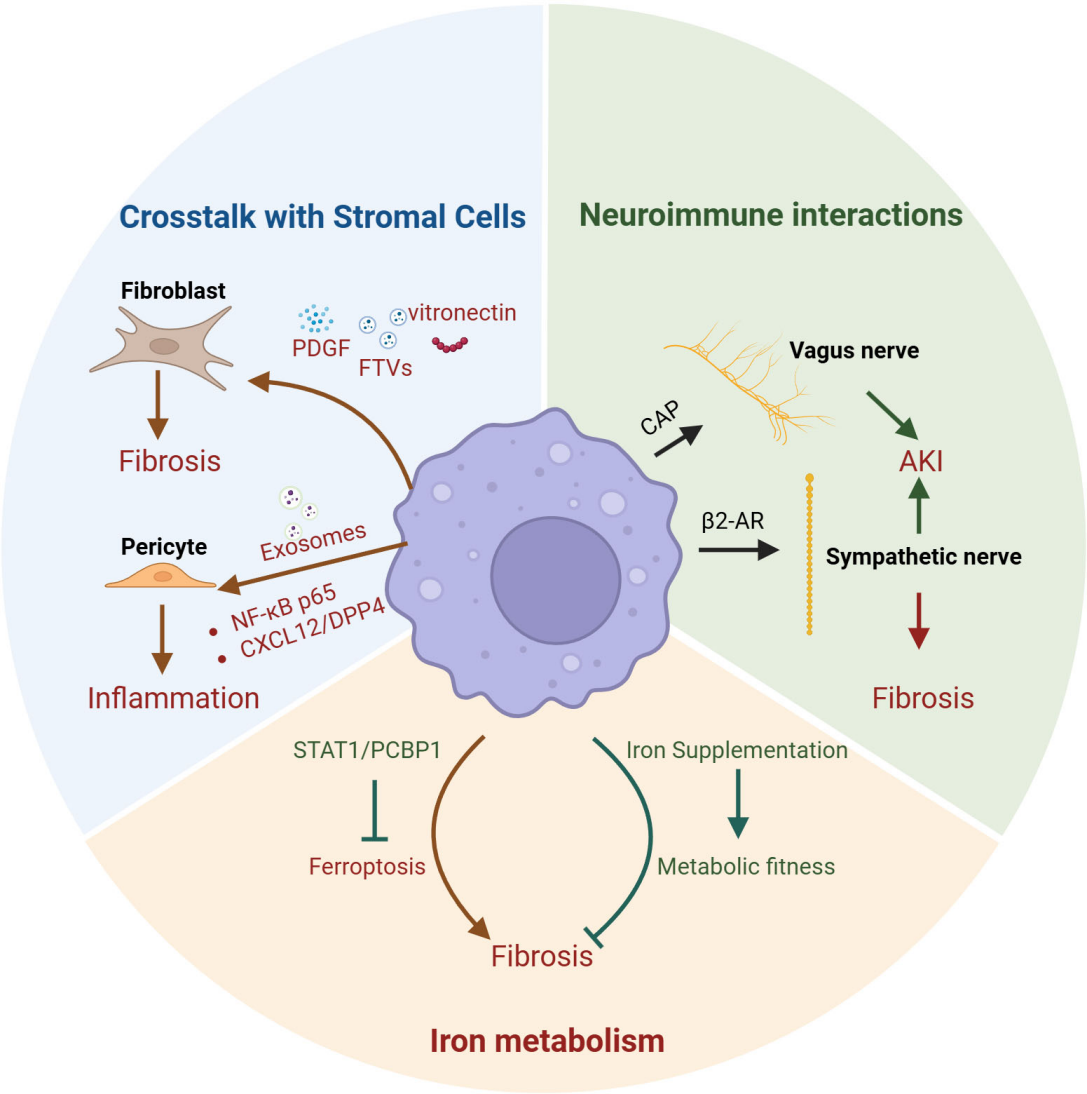
Macrophages non-canonical functions in kidney. The non-canonical functions of macrophages in the kidney also encompass crosstalk with stromal cells, iron metabolism, and neuroimmune interactions. Kidney macrophages promote fibrosis by secreting PDGF, FTVs, and vitronectin to activate fibroblasts; additionally, they can release exosomes that act on pericytes via the NF-κB p65 and CXCL12/DPP4 pathways to enhance inflammation. Therapeutic strategies such as iron supplementation in macrophages or inhibition of macrophage ferroptosis through the STAT1/PCBP1 axis have both been shown to attenuate renal fibrosis. Moreover, macrophages can modulate the vagus nerve through the CAP or influence sympathetic innervation via β2-AR signaling, thereby either alleviating acute kidney injury (AKI) or exacerbating renal fibrosis. PDGF, platelet-derived growth factor; FTVs, filopodial tip vesicles; NF-κB p65, Nuclear Factor kappa-light-chain-enhancer of activated B cells; CXCL12, the CXC motif chemokine ligand 12; DPP4, dipeptidyl peptidase 4; CAP, cholinergic anti-inflammatory pathway; β2-AR, β2-adrenergic receptor; IRI, ischemia–reperfusion injury; AKI, induced acute kidney injury; UUO, Unilateral Ureteral Obstruction.

Kidney macrophages, owing to their high heterogeneity and the complexity of the kidney microenvironment, exhibit non-canonical functions (**Figure 2**).

#### 3.7.1 Crosstalk with stromal cells

Stromal cells are defined as all non-epithelial and non-endothelial cells within an organ, such as fibroblasts, vascular smooth muscle cells (VSMCs), and pericytes *etc.* (103). The crosstalk between kidney macrophages and these cellular populations is of paramount significance. The interaction between kidney macrophages and fibroblasts is pivotal for both maintaining kidney homeostasis and driving disease progression (104). Macrophages can produce PDGF to stimulate fibroblast migration and proliferation, whereas fibroblasts can secrete CSF1 to promote macrophage migration, differentiation, and survival (105). In unilateral IRI–induced AKI, kidney macrophages can establish a vitronectin (VTN)–enriched extracellular microenvironment, which activates fibroblasts via integrin αvβ5– and Src kinase–mediated signaling, thereby promoting kidney fibrosis (106). In DKD, kidney macrophages can secrete filopodial tip vesicles (FTVs) enriched in IL-11, which can initiate fibroblast transdifferentiation and induce kidney interstitial fibrosis (107). Using single-cell sequencing technology, researchers identified a macrophage subset—monocyte-derived extracellular matrix remodeling-associated macrophages (EAMs)—that activates fibroblasts via insulin-like growth factor (IGF) signaling, thereby contributing to kidney fibrosis (108). Mural cells encompass VSMCs and pericytes, whereas mesangial cells represent specialized pericytes residing within the glomerulus (103). Kidney macrophages are associated with the progression of renal artery stenosis (RAS), suggesting a potential interaction between macrophages and VSMCs (109, 110). Exosomes secreted by high glucose (HG)-stimulated macrophages can disrupt the normal architecture of mesangial cells by mediating inflammatory responses through the Nuclear Factor kappa-light-chain-enhancer of activated B cells (NF-κB) p65 signaling pathway (111). Moreover, in lupus nephritis (LN), kidney macrophages can mediate mesangial cell proliferation and migration through the CXC motif chemokine ligand 12 (CXCL12)/dipeptidyl peptidase 4 (DPP4) axis, suggesting that targeting macrophages to suppress mesangial cell proliferation and migration may represent a potential strategy to delay the progression of LN (112).

#### 3.7.2 Iron metabolism

Inhibition of iron dependent ferroptosis in kidney macrophages through the STAT1/Poly(rC)-binding protein 1 (PCBP1) axis can attenuate kidney fibrosis (113). Notably, iron supplementation can restore the labile iron pool (LIP) in kidney macrophages, leading to reduced oxidative stress and pro-inflammatory cytokine levels and suppressed TGF-β-driven fibrotic response of macrophages (44, 114). These findings highlight the limitations of the traditional view that focuses solely on the detrimental effects of iron overload, and underscore the substantial therapeutic potential of dynamically regulating macrophage iron metabolism.

#### 3.7.3 Neuroimmune interactions

Kidney macrophages are capable of interacting with the nervous system, thereby mediating neuroregulation in the kidney. Under physiological conditions, KRMs contribute to the regulation of the kidney sympathetic nervous system (42). In pathological conditions, the sympathetic neurotransmitter norepinephrine (NE) has been shown to promote macrophage polarization, with β2-adrenergic receptor (β2-AR)–G stimulatory protein α-subunit (Gαs) signaling, in UUO mouse models (115). In IRI-induced AKI, sympathetic signaling via the β2-AR/protein kinase A (PKA) pathway activates T cell Ig and mucin domain 3 (Tim3) ^+^ macrophages within the kidney, thereby mitigating kidney injury (105). On the other hand, vagus nerve stimulation (VNS) attenuates IRI-induced AKI through the cholinergic anti-inflammatory pathway (CAP), acting via the spleen and peritoneal macrophages, thereby underscoring the substantial potential of extrarenal macrophages in neuroimmune interactions towards kidney diseases (116, 117).

## 4 Recently established paradigm of kidney macrophage based on single-cell and spatial transcriptomics

With the advance of single-cell and spatial transcriptomic technologies, the phenotypic, spatial, and functional heterogeneity of kidney macrophages has been increasingly recognized (42, 53, 118).

Single-cell and spatial transcriptomic analyses revealed that KRMs exert beneficial functions in responding to immune challenges and in maintaining the homeostasis of the local microenvironment. V-domain Ig suppressor of T cell activation (VISTA)^+^ KRMs take part in the clearance of apoptotic cells and suppression of excessive T cell activation, thereby mitigating overactive inflammatory responses, in IRI-induced AKI (119). In addition, the progressive decline in the number of KRMs during the course of DKD suggests their involvement in the pathophysiological processes of the disease (23).

Single-cell and spatial transcriptomic analyses also revealed that MDMs are recruited to the sites of injury when kidney damage occurs and drive the progression of disease. Blockade of this recruitment process confers significant protective effects. S100A8/A9^+^ MDMs infiltrate the kidney within hours after AKI onset, initiating and amplifying inflammatory responses (120). In the early phase of AKI, MDMs and neutrophils exhibited pronounced accumulation within the outer stripe of the outer medulla, indicative of the establishment of a pro-inflammatory microenvironment (108). In DKD patients, distinct MDM subsets exhibit spatial specialization, with TREM2^+^ and S100A4^+^ MDMs enriched in glomerular and tubular regions, whereas MRC1^+^ MDMs predominate in the tubulointerstitium (121), providing a framework for future spatially investigations.

In addition, KRMs also comprise specific subsets that contribute to disease progression. In IgAN, the high expression of CCL2 and CX3CR1 in KRMs aids active recruitment of monocytes and amplification of inflammatory responses. Moreover, KRMs exhibit marked metabolic reprogramming, with significant enrichment of the Notch signaling pathway and pathways regulating glycolysis, fatty acid metabolism, and amino acid metabolism, directing KRMs towards a pro-inflammatory phenotype in IgAN (64). In both Immune Checkpoint Inhibitor-associated nephrotoxicity (ICI-AN) and polycystic kidney disease, KRMs act as drivers of disease progression (122, 123).

Conversely, MDMs also comprise specific subsets that exert protective functions under pathological conditions. A distinct MDMs subset expressing MMP-12 emerges exclusively during the reparative phase of injury resolution and is implicated in tissue remodeling (124). Arg1^+^ MDMs, which accumulate in the kidney cortex, have been implicated in promoting tissue repair during AKI recovery (125).

In summary, single-cell and spatial transcriptomic technologies have elucidated the question of “which macrophage subtypes are located where, in proximity to which cell types, and what functions they exert,” providing multidimensional evidence that offers novel perspectives for future microenvironment-targeted interventions and the selective modulation of specific macrophage subpopulations.

## 5 Macrophage-based therapeutic strategies

Building upon above foundation of kidney macrophages, an expanding body of work has underscored the therapeutic potential of targeting macrophage function. Accordingly, the following section will introduce emerging macrophage-based therapeutic strategies aimed at modulating their activity to mitigate kidney injury and fibrosis (**Table 1**).

**Table 1:** Macrophage-based therapeutic strategies.

NO.	Name	Anti-inflammation/anti-fibrosis/macrophage based cell therapies	Main signal pathway	Kidney disease	Reference
1	Emapticap Pegol (NOX-E36)	anti-inflammation	CCL2/CCR2	DKD	(61)
2	CCX140-B	anti-inflammation	CCL2/CCR2	DKD and FSGS	(126, 127)
3	Fostamatinib	anti-inflammation	Syk-JNK	IgAN and ANCA-associated vasculitis	(128, 129, 141)
4	extracellular vesicles-IL-10	anti-inflammation	mTOR	AKI	(130)
5	Metformin	anti-fibrosis	AMPK	CKD	(86, 133, 134)
6	CAR-M	macrophage based cell therapies	IL-4 signalling pathway	AKI	(137)
7	*macrophages overexpressing CPT1a*	macrophage based cell therapies	fatty acid oxidation	*CKD*	(138, 139)
8	*TREM2 macrophages*	macrophage based cell therapies	PI3K-AKT	CKD	(140)

### 5.1 Anti-inflammation and anti-fibrosis

Pharmacologic inhibition of the CCL2/CCR2 axis is a viable strategy for modulating macrophage-driven inflammation. Emapticap Pegol (NOX-E36), a CCL2 inhibitor, has demonstrated efficacy in reducing proteinuria in patients with DKD (61). Similarly, clinical trials involving CCX140-B, a CCR2 antagonist, have reported decreased proteinuria in patients with DKD and focal segmental glomerulosclerosis (FSGS), suggesting a potential renoprotective effect (126, 127). These agents act by attenuating macrophage recruitment and activation, thereby disrupting inflammatory amplification loops.

Fostamatinib, an inhibitor of spleen tyrosine kinase (Syk), has shown therapeutic benefit in patients with IgA nephropathy and ANCA-associated vasculitis, partly by limiting macrophage infiltration. (128, 129).

Moreover, Extracellular vesicles based IL-10 delivery to kidney macrophages mitigates IRI-induced AKI, possibly through downregulation of the mTOR pathway (130).

Metformin, widely prescribed as a first-line agent for type 2 diabetes mellitus (T2DM) (131), has demonstrated potential renoprotective effects in large-scale cohort studies (132). Mechanistically, metformin activates AMP-activated protein kinase (AMPK), leading to the suppression of TGF-β expression (86, 133). This pathway mitigates macrophage polarization toward the M2 phenotype, thereby decelerating fibrotic remodeling in CKD. In a murine model of calcium oxalate (CaOx) crystal-induced kidney injury, metformin was shown to attenuate fibrosis and improve kidney function by modulating macrophage activation (134). However, high-dose metformin has been associated with deleterious effects in AKI, likely due to excessive neutrophil recruitment (135).

In addition, macrophages regulate ECM remodeling by modulating MMPs and tissue inhibitors of metalloproteinases (TIMPs), which inhibit MMP activity, leading to ECM accumulation and fibrosis (136).

### 5.2 macrophage-based cell therapies

Cell-based therapies have emerged as a promising frontier in clinical implications, and macrophage-based strategies are gaining substantial traction within kidney research. Researchers have developed a CAR-M capable of recognizing TNF and activating the intracellular IL-4 signaling pathway, thereby conferring anti-inflammatory functions in IRI-induced AKI (137). Genetically engineered macrophages overexpressing carnitine palmitoyltransferase 1a (CPT1a) exhibit augmented phagocytic activity by facilitating fatty acid oxidation and extracellular matrix clearance, suppressing pro-fibrotic cytokine release in CKD (138, 139). In addition, adoptive transfer of macrophages engineered to overexpress Triggering Receptor Expressed on Myeloid Cells 2 (TREM2) enhance the anti-inflammatory phenotype of kidney macrophages by PI3K/protein kinase B (AKT) pathway, resulting in reduced fibrosis and improved kidney function in CKD (140).

In summary, a substantial body of research has already focused on targeting kidney macrophages, aiming to modulate their polarization, phenotype, and function to achieve anti-inflammatory and anti-fibrotic effects, as well as to develop macrophage-based cell therapies, thereby attenuating disease progression.

## 6 Future directions and challenges

With ongoing technological innovation, our understanding of macrophage biology has deepened considerably. The advent of single-cell and spatial transcriptomics has revealed extensive cellular heterogeneity within macrophage populations, prompting the development of increasingly nuanced classification systems (118). However, the absence of methodological standardization and limited cross-platform reproducibility have impeded the establishment of universally accepted criteria for macrophage subset definition, particularly in kidney research.

Single-cell and spatial transcriptomic approaches are not without limitations. Investigators often classify macrophages according to their specific research objectives, yet no unified standard currently exists. This lack of standardization frequently results in discrepancies and even contradictory findings across different studies. For example, in the context of AKI, different investigators have reported diametrically opposed roles for Arg1^+^ MDMs, with some studies attributing to them pro-inflammatory functions, whereas others suggest a pro-repair phenotype (124, 125). Moreover, divergent trends in the overall shift toward M2-like macrophages have been reported in late-stage DKD across different studies (23, 121). Clarifying these differences could provide valuable insights for the development of precision strategies to target specific macrophage subsets.

Future research should prioritize the development of standardized analytical pipelines, physiologically relevant *in vitro* models, and rigorous functional validation strategies. These advances will be essential to fully exploit the therapeutic potential of macrophage heterogeneity in kidney disease.

## 7 Conclusion

The growing body of evidence underscores the indispensable role of macrophages in the physiological maintenance and pathological progression of kidney diseases. Macrophage subpopulations, shaped by their microenvironment, demonstrate remarkable functional plasticity in modulating immune responses, mediating tissue injury, promoting regeneration, and driving fibrosis. Technological advancements such as single-cell and spatial transcriptomics have propelled our understanding of kidney macrophage heterogeneity, yet significant gaps remain regarding the functional validation and therapeutic translation of these insights. Future research could emphasize the development of standardized classification criteria, robust *in vitro* modeling systems, and subset-specific targeting strategies to optimize macrophage-based therapies. Ultimately, harnessing the therapeutic potential of macrophages represents a promising avenue to mitigate kidney injury, prevent fibrosis, and improve outcomes in patients with kidney diseases.
